# Unraveling heterogeneity of consumers’ food choice: Implications for nutrition interventions in eastern India

**DOI:** 10.1016/j.gfs.2021.100497

**Published:** 2021-03

**Authors:** Marie Claire Custodio, Jhoanne Ynion, Arindam Samaddar, Rosa Paula Cuevas, Suva Kanta Mohanty, Anindita Ray (Chakravarti), Matty Demont

**Affiliations:** a International Rice Research Institute, Los Baños, Laguna, Philippines; b Kalinga Institute of Industrial Technology, KIIT School of Management, Bhubaneswar, India; c Institute of Rural Management Anand (IRMA), Anand, Gujrat, India; d Department of Food & Nutrition, Maharani Kasiswari College, University of Calcutta, India

**Keywords:** Gastronomic system, Food system, Food choice, Nutrition, Consumers, Eastern India

## Abstract

Understanding heterogeneity of consumers' food choice is critical in formulating tailored nutrition interventions. To illustrate this, we survey urban and rural consumers from low- and middle-income households in eastern India to unravel five sources of heterogeneity (5 Ws) in gastronomic systems that affect diets: (i) socioeconomic characteristics of the target population (*who*); (ii) food environments (*where*); (iii) eating occasions (*when*); (iv) consumed dishes (*what*); and (v) ingredient attributes and consumer attitudes towards food (*why*). Diets in eastern India are predominantly starch-based featuring infrequent intake of fruits and vegetables. Accounting for heterogeneity in gastronomic systems can help policy makers and nutritionists develop more targeted nutrition interventions, which can aid in the development of planetary health diets in various contexts around the world.

## Highlights

• Nutrition interventions need to account for heterogeneity of consumers' food choice.
• We survey a target population of low- and middle-income households in eastern India.
• Diets are predominantly starch-based with infrequent intake of fruits and vegetables.
• We unravel five sources of heterogeneity in gastronomic systems that affect diets.
• Accounting for heterogeneity can help better targeting nutrition interventions.

## 1 Introduction

The EAT-Lancet Commission, a highly influential multi-disciplinary think tank of leading scientists from 16 countries, recently published an urgent call for food system transformation to catalyze a dietary shift towards “planetary health diets”; i.e., diets that are mainly composed of plant-based instead of animal-based food sources and that aim at improving both human health and environmental sustainability (Willett et al., 2019). It emphasizes the need to integrate nutrition and sustainability into a global agenda for food system transformation. The role of nutrition in the food systems paradigm is crucial because it is not only one of the Sustainable Development Goal (SDG) outcomes (i.e. good health and well-being) but also a means to achieve several outcomes (Development Initiatives, 2017). However, the success of nutrition interventions ultimately hinges on people—consumers’ behavior as influenced by their physiological and nutritional needs, their socio-demographic contexts, their hedonic motivations, and their attitude and beliefs towards food (Cuevas et al., 2021; Haddad, 2020; Jain et al., 2014; Samaddar et al., 2020; Sheikh and Mohan, 2015; Shepherd, 1999). Instead of developing a blanket approach to nutrition intervention, policy makers and nutritionists need to be informed about the heterogeneity of the food choices the target population faces and makes such that targeted and segmented nutrition interventions can be planned, designed and implemented.

**Fig. 1:**
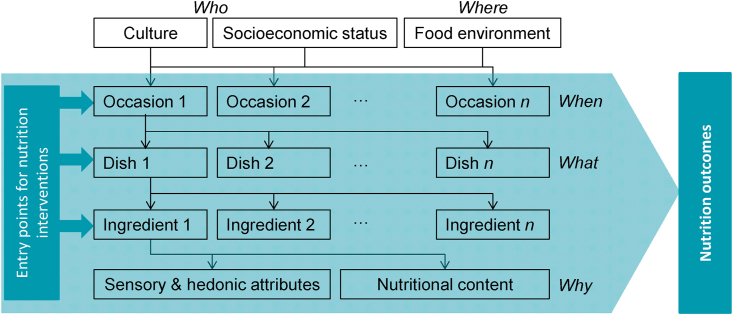
The five sources of heterogeneity (5 Ws) in the gastronomic system. *Source*: adapted from Cuevas et al. (2017; 2021) and Samaddar et al. (2020).

Consumers' food choice features five sources of heterogeneity, which can be captured through 5 Ws in the “gastronomic system” (Fig. 1; Cuevas et al., 2017; 2021; Samaddar et al., 2020). First, the gastronomic system is shaped by (i) the culture and socioeconomic context of a heterogeneous population of consumers (*who*); and (ii) a heterogeneous set of food environments where consumers access and consume food (*where*). The gastronomic system on its turn shapes consumers’ eating patterns and diets at three hierarchical levels: it gives rise to (iii) a heterogeneous set of eating occasions during which food is typically consumed (*when*); these occasions in their turn determine (iv) a heterogeneous set of dishes that are purchased, prepared and consumed (*what*), which command (v) a heterogeneous set of ingredients and ingredient pairings that carry sensory, hedonic, cooking, convenience, and nutritional attributes and are subject to heterogeneous consumer attitudes and motivations (*why*). The three levels of the gastronomic system—occasions, dishes and ingredients—provide entry points for nutrition interventions in food environments and the 5 Ws can help better articulating and targeting those interventions to nudge the target population towards healthier diets and generate nutrition and health outcomes.

To illustrate the importance of taking into account heterogeneity of consumers' food choice, it is useful to focus on India, a large country featuring a rich cultural heritage and diversity of food choice (Samaddar et al., 2020). Similarly to the rest of South Asia, India has benefitted from the gains of the Green Revolution with significant improvement in staple cereal production since the 1970s (Pingali, 2015). However, nutritional challenges remain despite improvement of the food system in increasing availability of food (Allen and de Brauw, 2018). The country continues to suffer from malnutrition and features the highest estimated number of undernourished people in the world. Sharma et al. (2020) demonstrate through a large representative sample of households how unhealthy Indian diets are and how much they differ from the planetary health diets recommended by the EAT-Lancet Commission (Willett et al., 2019). Indian diets are rich in cereals, but poor in proteins, fruits, and vegetables. For example, with the exception of wealthy urban consumers, fruit consumption among Indian consumers is found to be 40% lower in terms of calories than the recommended amount by EAT-Lancet. The public distribution system, which has highly subsidized rice and wheat consumption, may have contributed to such a phenomenon. However, several constraints are likewise identified which include consumers’ access and affordability of nutritious food, particularly of fruits and vegetables, as well as lack of awareness regarding the health benefits of consuming the latter (Choudhury et al., 2020). Affordability of fruits and vegetables in rural areas is by far lower than in urban areas, due to income constraints and low wages (Choudhury et al., 2020; Sachdeva et al., 2013). Since poor households usually have many dependents and high non-food expenditures, these households find it difficult to afford nutritious diets (Raghunathan et al., 2020). Job loss due to economic downturn further constraints the marginalized section in the urban areas to afford nutritious food. It has been argued that nutrition deficiencies among urban households decrease with the rise in income (Rao et al., 2018) and that affordability is a key barrier in increasing fruit and vegetable intake (Finzer et al., 2013; Surendran et al., 2020).

In the eastern region of India (i.e., the states of Odisha and West Bengal), people mainly consume starchy staples (e.g., rice and potato), which are rich in energy content but nutritionally poor (Panda et al., 2015). A substantial share of the population in both Odisha and West Bengal is undernourished, as indicated by measures of chronic energy deficiency (Das and Bose, 2015). Although diets of urban consumers in the region, particularly the middle- and high-income households, have been found to be improving and diversifying through inclusion of higher valued food items (Ali et al., 2010; Brokaw and Lakshman, 1995; Khush, 2005; Landes and Gulati, 2004; Mottaleb et al., 2018a, 2018b; Pingali and Khwaja, 2004), the poor are still largely left out of these trends. The poor have been reported to lack access to more nutritious food, such as milk and vegetables (Bhattacharya, 2013; Chand and Gartia, 2016).

The Government of India (GoI) has implemented nutrition interventions—both nutrition-sensitive and nutrition-specific—to address the triple burden of malnutrition (i.e., undernutrition, micronutrient deficiency, and overnutrition). Particularly to improve consumption of fruits and vegetables at the household level, various agencies of the GoI initiated nutrition-sensitive interventions which tackle both the demand and supply (Sachdeva et al., 2013). On the demand side, some examples of interventions are dietary advice and counselling services in primary health care centers, engagement with media and institutions and establishing a dedicated website (healthy-india.org) to promote the health benefits of fruits and vegetables. On the supply side, some examples of nutrition-sensitive interventions are provision of technical advice to farmers, establishment of nurseries and tissue culture units for production and distribution of seeds, encouragement of organic farming, and issuance of recommendations on how to rejuvenate old orchards, and to manage water sources (Sachdeva et al., 2013). The GoI has likewise implemented nutrition-specific interventions intended for lactating women and children (e.g., Integrated Child Development Scheme), rural populations (e.g., National Rural Health Mission), and the poor (e.g., Targeted Public Distribution System, Mahatma Gandhi National Rural employment Guarantee Act) (Mohmand, 2012; Government of Odisha, 2015). Such nutrition-specific interventions target women and children because in the food and nutrition security agenda, maternal and children's health are considered as one of the foundations of good nutrition of a nation (UNICEF, 2016). However, while these interventions focus on improving the access to food and implementation of nutrition-specific programs on food, feeding practices and infection prevention, little information is available on the heterogeneity of consumers' food choice in India, and how this affects the optimal design of nutrition interventions.

The success of nutrition interventions crucially hinges on a thorough understanding of the five sources of heterogeneity (5 Ws) in the gastronomic systems of target populations. Therefore, in this article, we illustrate these sources of heterogeneity in a target population of urban and rural low- and middle-income households in eastern India. Accounting for heterogeneity in gastronomic systems can help policy makers and nutritionists develop more targeted nutrition interventions, which can aid in the development of planetary health diets in various contexts around the world.

## 2 Methodology

We define our target population as urban and rural low- and middle-income households in eastern India. We have identified these income classes to represent the economically-vulnerable segment of the population, which most of the time overlaps with the nutritionally-vulnerable one. Cuevas et al. (2021) developed a toolkit to capture diversity and drivers of food choice of a target population and identify entry points for nutrition interventions through a three-stage mixed methods approach: Stage 1—capturing diversity and cultural drivers of food choice; Stage 2—understanding consumer perceptions and drivers of food choice; and Stage 3—nudging behavioral change through interventions. Diversity and cultural drivers of food choice of our target population (Stage 1) were captured by Samaddar et al. (2020); here we focus on Stage 2 and validate and quantify the qualitative findings in Stage 1 through a consumer survey. Our consumer survey was succeeded by a behavioral experiment (Stage 3) to test the impact of a nutrition intervention on households’ food choice planning (Demont et al., 2019).

### 2.1 Data collection and sampling

**Fig. 2:**
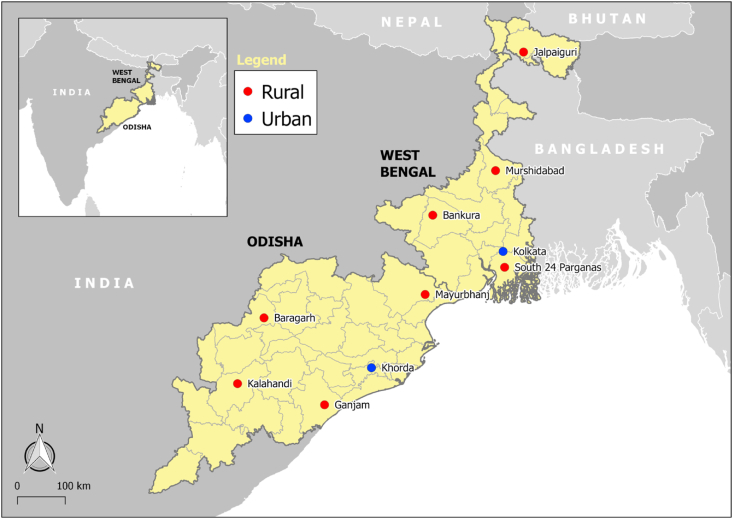
Map of survey sites in eastern India. *Notes*: Total sample size = 501 (Odisha: 251; West Bengal: 250; urban: 253; rural: 248; middle income: 108; low income: 393; urban Odisha: 127; rural Odisha: 124; urban West Bengal: 126; rural West Bengal: 124; middle-income Odisha: 51; low income Odisha: 200; middle income West Bengal: 57; low income West Bengal: 192).

A consumer survey with 501 households of the target population was conducted through door-to-door interviews in urban and rural districts in Odisha and West Bengal from November to December 2017 (Fig. 2). The geographic scope in each state included the capital city and four rural districts. The capital city was selected to represent the urban zones in the state (i.e., Bhubaneswar in Odisha and Kolkata in West Bengal). Four rural districts in each state were selected based on nutritional status (i.e., proportion of the population that is under or over-nourished, based on body mass index) using the results of the National Family Health Survey 2015–2016 (International Institute for Population Sciences, 2015), the population size based on the 2011 population census (Census Organization of India, 2017), and the geographic spread of the rural districts. A multi-stage sampling procedure was implemented. In the first stage, we implemented a stratified random sampling where city or rural districts were stratified into geographical zones (i.e., north, south, east, and west). Primary sampling units (PSUs) were then randomly selected in each zone. In the second stage, we applied systematic sampling of households following the right-hand rule from a starting point with a sampling interval of three households. Screening questions in the questionnaire were then used to identify possible qualified household members. One of the key qualifying criteria for respondent selection is active involvement in grocery purchase decision-making or active involvement in cooking/meal preparation/meal planning for the household. In cases where more than one household member met the selection criteria, the qualified person who had the most recent birthday was selected. A pen-and-paper structured questionnaire was used to elicit information from the respondents. To assess households' diet diversity, key information regarding their consumption behavior were collected, particularly the dishes consumed during different daily eating occasions and the corresponding frequency of consumption of each dish in a typical month. Consumers’ attitude towards food was assessed by asking respondents to provide an importance rating score for eleven pre-defined food quality attributes. Purchase-related questions were likewise asked to gain insights on how consumers interact with their food environment. The sampling and data collection methodology is described in more detail in the accompanying *Data in Brief* article (Ynion et al., submitted).

### 2.2 Data analysis

Sampling weights were used in the analysis to reduce sampling bias and to account for possible over-representation of certain income classes in urban and rural districts of each state (Solon et al., 2013).

Consumption behavior and attitudes were assessed through diet diversity and through factor and cluster analyses, respectively. Diet diversity (Fig. 5) and the average frequency of consumption were computed based on each household's frequency of consumption of dishes that they consume in a typical month (Table 3; Table 4). For every eating occasion (i.e., breakfast, AM snacks, lunch, PM snacks, dinner), each respondent was asked to identify the dishes their household consumes in a typical month and describe the frequency of consumption using a scale. We estimated dietary diversity by computing households' consumption frequency of the dishes from different food groups consumed in a typical month; the serving portions of dishes (amount of serving on a plate) were not captured in the survey. Spearman rank correlation, a non-parametric test (Akoglu, 2018), was used to test the strength and direction of the association between the frequency of purchase and distance of store type (Table 2). To examine popular dish pairings in Fig. 6, the frequencies of co-occurrence of dishes (i.e., the number of times each dish is being paired with another dish at an eating occasion) were computed and visualized through heat maps. Consumers' attitude towards food and diets was assessed through exploratory factor analysis (EFA) (Fig. 8) (Hair et al., 2009), resulting in a seven-factor solution. Consumer segmentation based on the themes derived from the EFA was done through non-hierarchical K-means clustering (Fig. 9), resulting in a three-cluster solution. The specific approaches to each of the aforementioned analyses and software used are described in more detail in the accompanying *Data in Brief* article (Ynion et al., submitted).

**Table 1:** Socio-demographic profile of respondents.

	All	Odisha	West Bengal
Sample size	501	251	250
Urban	50%	50%	51%
Rural	50%	50%	49%
Male	29%	38%	21%
Female	71%	62%	79%
Main purchase decision maker and involved in cooking/meal planning	84%	81%	87%
Main purchase decision maker and not involved in cooking/meal planning	6%	10%	2%
Not involved in purchase decision-making but involved in cooking/meal planning	10%	8%	11%
Low-income households	78.4%	79.7%	77.0%
Middle-income households	21.6%	20.3%	23.0%
Monthly household income, mean			
Urban households (INR)	14,330	14,989	13,667
Urban households (USD)^a^	222	232	212
Rural households (INR)	6,243	5,861	6,627
Rural households (USD) a	97	91	103
Age, mean (in years)	39	37	40

a Foreign exchange rate in December 2017 was 1 US$ = 64.5 INR.

**Table 2:** Consumers’ usual place of food purchase in urban and rural districts of Odisha and West Bengal.

Usual place of purchase (% of respondents)		All	Odisha	West Bengal	Urban	Rural	Middle income	Low income	Correlation: distance and frequency of purchase
Meat/fish/poultry	Weekly market	98	98	98	100	96	99	98	−0.38**
Vegetable	Weekly market	97	97	98	100	94	97	97	−0.34**
Fruits	Weekly market	94	96	91	98	89	96	93	−0.29**
Rice	Local grocery store	73	76	70	80	66	75	72	0.17**
	Weekly market	21	19	24	14	29	22	21	0.05
Other cereals	Local grocery store	78	73	83	89	66	79	77	−0.04
	Weekly market	19	24	15	6	33	18	20	0.28**

Other food products	Local Grocery store	75	70	81	81	70	69	77	−0.04
	Weekly market	22	28	16	15	29	27	21	0.16**
Distance from house (median, in meters)									
Weekly market		1000	2000	500	500	2000	500	1000	
Local grocery store		300	200	500	300	250	300	300	

*Notes*: Spearman's correlation was used to test the strength and association between frequency of purchase of and distance of store type from the house since data for both variables are not normally distributed. **Correlation is significant at 0.01 level.

**Table 3:** Most frequently consumed dishes in urban and rural districts of Odisha during daily dining occasions.

Occasion	Dish classification	Urban	Rural
Breakfast	Starch-based	Steamed rice (28); Toasted bread (24); *Paratha*^a^ (12)	Boiled corn (28); *Dalia* (wheat porridge) (28); *Fry chura* (flattened rice) (28)
	Fruit-based	None	*Chutney* (Condiment – tomato or cucumber or other fruit) (16)
	Non-vegetarian	Chicken *do pyaza* (chicken with onions) (20); Egg omelet (7)	Egg omelet (9); Boiled dry fish (9)
	Dairy	None	None
	Pulses	*Dalma*^b^ (28); Almond (20); *Ghugni* (chickpeas) (6)	*Dal*^b^ (13); Vada (fried snacks of pulses) (8); *Ghugni* (chickpeas) (5)
	Vegetables	*Santula* (fried vegetable dish) (10)	Vegetable curry (20); Vegetable dish (Cauliflower) (20); Any veg dish (14)
AM Snacks	Starch-based	Biscuit (16); *Moori*^c^ (6)	*Moori*^c^ (19); Biscuit (13)
	Fruit-based	Any fruit (5)	Any fruit (4)
	Non-vegetarian	None	None
	Dairy	None	None
	Pulses	Roasted peanut (5)	Roasted peanut (13)
	Vegetables	None	None
Lunch	Starch-based	Steamed rice (27); *Panta bhat*^d^ (7); *Papad* (flat circular pieces made from pulse flour) (7); *Chapati*^a^ (7)	Steamed rice (25); *Panta bhat*^d^ (16); Fried potato (14); *Chapati*^a^ (14)
	Fruit-based	*Khatta* (Condiment – tomato or cucumber or other fruit) (6)	*Khatta* (Condiment – tomato or cucumber or other fruit) (11)
	Non-vegetarian	Fish curry (6); Egg curry (3); Chicken curry (3); Prawn *malai* curry (3); Mutton curry (2)	Crab curry(18); Fish curry (10); Egg curry (10); *Mangsher jhol* (mutton curry) (9); *Murgir jhol* (chicken curry) (8)
	Dairy	*Raita* (sour curd) (5); *Paneer* curry (cottage cheese) (2)	*Raita* (sour curd) (13); *Paneer* curry (cottage cheese) (6)
	Pulses	*Dal*^b^ (22); *Dalma*^b^ (16); Dal (black gram) (10)	*Dal*^b^ (18); *Dalma*^b^ (12); *Ghugni* (chickpeas) (12)
	Vegetables	Mixed vegetable (8); Mixed vegetable curry (8); *Besara rai* (Type of veg curry in mustard paste) (7); *Saag* (drumstick leaves) (5)	*Saag* (drumstick leaves) (14); Mixed vegetable (13); Mixed vegetable curry (12)
PM snacks	Starch-based	Biscuit (13); *Moori*^c^ (8); Noodles (7)	Sago (20); *Moori*^c^ (19)
	Fruit-based	None	None
	Non-vegetarian	None	None
	Dairy	None	*Paneer* (cottage cheese) (10); *Malpua* (fried flour cake with sweet milk filling) (4)
	Pulses	*Vada* (fried snacks of pulses) (6); *Ghugni* curry (5)	Dalma (curry) b (28); Dried peas curry (15); *Chola buta* (Chickpea) (10); Almond nuts (10)
	Vegetables	None	Cauliflower *Pakoda*^e^ (10); Eggplant *Pakoda*(10)
Dinner	Starch-based	*Gola Roti* (pancake) (22*); Panta bhat*^d^ (20); Steamed rice (14)	Steamed Rice (24); Fried potato (20); *Ragi* porridge (Finger millet) (19)
	Fruit-based	*Chutney* (Condiment – tomato or cucumber or other fruit) (3)	*Chutney* (Condiment – tomato or cucumber or other fruit) (12)
	Non-vegetarian	Fish curry (4); Egg curry (3); *Murgir jhol* (chicken curry) (3)	Fish curry (10); Egg curry (9); *Murgir jhol* (chicken curry) (7)
	Dairy	*Paneer* curry (cottage cheese) (3)	*Paneer* curry (cottage cheese) (7)
	Pulses	*Dalma*^b^ (17); *Dal*^b^ (13), *Rajma (*Kidney bean) curry (9)	*Dal*^b^ (16), *Rajma (*Kidney bean) Curry (12); *Dalma*^b^ (10)
	Vegetables	*Santula* (fried vegetable dish) (10); Mixed vegetable (9)	*Saag* (20); *Santula* (fried vegetable dish) (12); Mixed vegetable (12)

*Notes:* The numbers in ( ) refer to the monthly average frequency of consumption where average frequency = sum of frequency/no. of mentions. For each dish that the respondent mentioned that they consume in a typical month, we asked them to use a scale to indicate the frequency of consumption. A midpoint value was then assigned as: everyday = 28, 4–6 times per week = 20, 2–3 times per week = 10, Once a week = 4, 2–3 times per month = 2.5, Once a month = 1. Sum of frequency of each dish was computed.

a *Paratha, chapati,* and *roti* are different types of flatbread.

b *Dal* refers to dried pulses (i.e., lentils, peas, beans). There are many versions and terms used to refer to *Dal* dishes (e.g., *Dalma*) depending on the ingredients and type of cooking.

c *Moori* is puffed rice which can be mixed with oil, spices, onions, tomato.

d Boiled rice fermented in excess water is referred to as *Pakhala bhat* in OD.

e *Pakoda* is deep fried dough made from Bengal gram flour. We classify it either as veg or pulse, depending on the ingredient added in the dough. We classified it as starch for the responses without specific veg or pulse or as plain mixture.

**Table 4:** Most frequently consumed dishes in urban and rural districts of West Bengal during daily dining occasions.

Occasion	Dish classification	Urban	Rural
Breakfast	Starch-based	*Moori*^a^ with *pakoda*^b^ (28); *Samosa* (flour envelopes with vegetable filling) (20); Fried Potato (19)	*Aloo chokha* (mashed potato with bengal gram) (20); *Moori*^a^ (19); Boiled potato (19)
	Fruit-based	Any fruit (14); Cucumber (10)	Any fruit (13); *Chutney* (condiment – tomato or cucumber or other fruit) (10); Cucumber (6)
	Non-vegetarian	Boiled Egg (12)	Boiled Egg (13)
	Dairy	Sweets (10); *Malpua* (fried flour cake with sweet milk filling) (4)	Sweets (4);
	Pulses	*Dal*^c^*(peas)*(12); *Chatua* (bengal gram) (9); *Kachori* (round flattened ball made of gram flour and moong dal) (7); *Ghugni* (dried peas in gravy) (7)	Dal (gram seeds) (28); *Chatua* (bengal gram) (11); *Chanachur*^e^ (10); *Dal*^c^ (10);
	Vegetables	*Torkari*^d^ (18); Fried *brinjal* (eggplant) (10); Vegetable dish (Cauliflower) (10)	*Torkari*^d^ (16); Onion *Pakoda*^b^ (14); Fried brinjal (11)
AM Snacks	Starch-based	*Paratha*^f^ (20); *Pakoda*^b^ (20); Steamed rice (20)	Steamed rice (21); Boiled potato (17); *Moori*^a^*masala* (12)
	Fruit-based	Any fruit (11)	Any fruit (28); Banana (10); Guava (10); Lemon (10)
	Non-vegetarian	None	Fish curry (14); Egg curry (9)
	Dairy	Sweets (8); Ice cream (9); *Chhanna* (cottage cheese) (5)	Sweets (10); *Chhanna* (cottage cheese) (3)
	Pulses	*Chanachur*^e^ (12); *Chatu Gola* (7); Dried Peas (6); *Rajma* (Kidney bean) curry (6)	*Dal*^c^ (15); *Chanachur*^e^ (10); *Rajma* (Kidney bean) curry (6)
	Vegetables	Vegetable Soup (10); Sprouts (2)	Vegetable curry (19); Sprouts (16); Gond Radish & potato (10); Eggplant *Pakoda* (10); Vegetable dish (Cauliflower) (10)
Lunch	Starch-based	Steamed rice (27); *Chapati*^f^ (13); Fried potato (10)	Steamed rice (27); *Chapati*^f^ (12); Dalia (wheat porridge) (10);
	Fruit-based	*Chutney* (condiment – tomato or cucumber or other fruit) (10); *Chat* (fruit salad) (6)	*Chutney* (condiment – tomato or cucumber or other fruit) (6)
	Non-vegetarian	Fish curry (17); Egg curry (10); Chicken *tandoori* (grilled) (10); Boiled egg (10)	Fish curry (9); Egg curry (7); Fried fish (6)
	Dairy	*Raita* (sour curd) (10); *Malai kazai* (milk dessert)(10); Yogurt (7); *Paneer* (cottage cheese) (7)	Yogurt (21); *Paneer* (cottage cheese) (3)
	Pulses	*Dal (Pakoda)*^c^^,^^b^ (20); *Dal*^c^ (19); *Bari* curry (sun dried lentil balls in spicy gravy) (8); Soybean curry (8)	Dal e (15); *Dal (Pakoda)*^c^^,^^b^ (10); Soybean curry (8); *Dhoka* (pulse) (8)
	Vegetables	*Torkari*^d^ (23); Mixed vegetable (19); *Saag* (drumstick leaves) (16)	*Torkari*^d^ (22); *Saag* (drumstick leaves) (11); Mixed vegetable (11)
PM snacks	Starch-based	*Bhelpuri* (sweet & sour puffed rice mix) (28); *Chapati*^f^ (20); *Moori*^a^ (17)	*Moori* with oil a (22); *Moori*^a^ (17); Biscuit (19); *Luchi* (deep-fried wheat dough) (19);
	Fruit-based	None	Cucumber (20)
	Non-vegetarian	Mutton (non-curry) (10); Egg roll (6); Fish chop (5)	Egg roll (4); Fish chop (2)
	Dairy	*Sandesh* (sweetened cottage cheese) (7); Ice cream (3); *Dahi vada* (fried lentil balls in yoghurt) (3)	*Sandesh* (sweetened cottage cheese) (4);
	Pulses	*Chanachur*^e^ (17); Roasted peanut (12); *Kachori* (round flattened ball made of gram flour and moong dal) (10)	*Chanachur*^e^ (12); Roasted peanut (11); Fried nuts (10)
	Vegetables	Vegetable *Pakoda*^b^ (9); Cauliflower *Pakoda*^b^ (1)	Vegetable Dish (11); Eggplant *Pakoda*^b^ (10); Vegetable *Pakoda*^b^ (7)
Dinner	Starch-based	Boiled potato (28); Steamed rice (19); Steamed cake (14)	Boiled potato (28); Steamed rice (19); *Panta bhat*^g^ (17)
	Fruit-based	*Chutney* (condiment – tomato or cucumber or other fruit) (9)	*Chutney* (condiment – tomato or cucumber or other fruit) (6)
	Non-vegetarian	Fish curry (13); Fried egg (10); Egg curry (8)	Beef curry (10); Fish curry (8); Egg curry (8)
	Dairy	*Simui payesh* (vermicelli with milk) (12); *Rosogollah* (cottage cheese balls in sugar syrup) (7); *Paneer* curry (cottage cheese) (5)	*Rosogollah* (cottage cheese balls in sugar syrup) (5); *Paneer* curry (cottage cheese) (4); *Raita* (sour curd) (4)
	Pulses	*Dal*^c^ (19); *Tadka* curry (6); *Rajma (*Kidney bean) curry (5)	*Chana* curry (chickpeas) (28); Soybean curry (20); *Dal*^c^ (14)
	Vegetables	*Torkari*^d^ (22); Vegetable curry (17); Fried *brinjal* (eggplant) (10); Vegetable dish (Cauliflower) (10)	*Torkari*^d^ (22); Vegetable curry (12); Fried *brinjal* (eggplant) (10); Vegetable salad (10); Vegetable dish (Cauliflower) (10)

*Notes:* The numbers in ( ) refer to the monthly average frequency of consumption where average frequency = sum of frequency/no. of mentions. For each dish that the respondent mentioned that they consume in a typical month, we asked them to use a scale to indicate the frequency of consumption. A midpoint value was then assigned as: everyday = 28, 4–6 times per week = 20, 2–3 times per week = 10, Once a week = 4, 2–3 times per month = 2.5, Once a month = 1. Sum of frequency of each dish was computed.

a *Moori* is puffed rice which can be mixed with oil, spices, onions, tomato.

b *Pakoda* is deep fried dough made from Bengal gram flour. We classify it either as veg or pulse, depending on the ingredient added in the dough. We classified it as starch for the responses without specific veg or pulse or as plain mixture.

c *Dal* refers to dried pulses (i.e., lentils, peas, beans). There are many versions and terms used to refer to *Dal* dishes (e.g., *Dalma*) depending on the ingredients and type of cooking.

d *Torkari* (*Tarkari*) is a type of vegetable curry.

e *Chanachur* is a spicy savoury snack with lentils and dry fruits.

f *Paratha, chapati,* and *roti* are different types of flatbread.

g Boiled rice fermented in excess water is referred to as *Panta bhat* in West Bengal.

### 2.3 Ethical considerations

The door-to-door survey with households was organized under the “Behavioral drivers of food choice in eastern India” project. The survey questionnaire obtained ethics approval from the International Rice Research Institute's (IRRI) Institutional Research Ethics Committee (IREC 18-001). Before the start of each interview, the respondents were informed that the survey is for research purposes, their participation is voluntary, and that all personal information will be kept confidential. The respondents gave verbal consent to be interviewed.

## 3 Results and discussion

### 3.1 Who: socioeconomic context of the target population

Differences in socio-economic contexts among the target population represent the first source of heterogeneity that policy and nutritionists need to account for in the design of targeted nutrition interventions (Fig. 1). The respondents were males and females between 18 and 64 years old from low- and middle-income households (Table 1). The monthly income ranges in the cities were INR15,000 (US$233) and below for low-income households and INR15,001 (US$233) to INR 85,000 (US$1318) for middle-income households. The income ranges in rural districts were INR7,000 (US$109) and below and INR7,001 (US$109) to INR50,000 (US$775) for low- and middle-income households, respectively. The income distribution of households from the India Human Development Survey-II (IHDS-II) 2011–2012 (Desai and Vanneman, 2018) was used as a reference for the income classification used in this study. As mentioned in the Methodology section, one of the main respondent's selection criteria was involvement in purchase decision-making of food products for the household. The target respondent may also be involved in cooking, meal preparation, or meal planning for the household (Table 1). The majority of respondents who qualified to be interviewed were females. But it is interesting to note that a sizeable share (almost 30%) of the respondents were male household members. This may indicate that nutrition-sensitive interventions should not only target the female household members (i.e., wife, grandmother) as nutrition-specific programs typically do, but should also include males who have involvement in food items purchase and in meal planning or cooking. Additional sources of consumer heterogeneity will be discussed in Section 3.5 in conjunction with consumer attitudes towards food.

### 3.2 Where: food environments

The food environments where food is accessed represent the second source of heterogeneity (Fig. 1). Table 2 reveals important heterogeneity in consumers’ physical access to various food products in both the urban and rural areas, income categories, and between the states. This heterogeneity partly explains the deficit in fruits and vegetables of eastern Indian diets (Sharma et al., 2020) and may be attributed to inaccessibility of nutritious foods such as fruits and vegetables by the poor (Bhattacharya, 2013; Chand and Gartia, 2016). Availability (i.e., whether a food item is present within a physical range) is one of the key elements of the food environment where one can see the interaction of physical distance between home and place of purchase (HLPE, 2017; Turner et al., 2018; Downs et al., 2020). Consistent with Finzer et al. (2013), Table 2 suggests that in eastern India, fruits and vegetables are typically purchased from weekly markets, the location of which is usually farther from home relative to the location of local grocery stores, where rice and other dry goods are commonly purchased (Table 2). The households surveyed tend to buy fruits and vegetables less often the farther the distance of the weekly market. These observations are particularly true for households without home gardens or community gardens and for fruits and vegetables sourced from other localities.

**Fig. 3:**
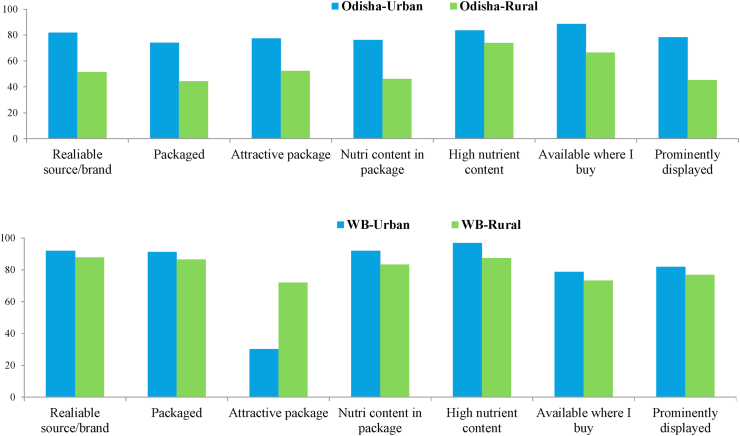
Attitude towards food purchase of urban and rural consumers in Odisha and West Bengal based on their importance rating of predefined attributes. *Note*: The values in the graph indicate the percent of respondents who gave a rating of 5 or 4 out of the 5-point Likert scale where 5 means extremely important and 1 means not at all important.

Promotion is another element of the food environment which refers to how a food item is presented, marketed, promoted and labeled to influence the desirability of food (Downs et al., 2020). Fig. 3 reveals perceptual differences of urban and rural consumers on the promotional aspect of food. Urban consumers tend to procure food from stores and attribute more importance to this food environment than rural consumers. This is not surprising because consumers in rural districts have closer proximity and access to the natural food environments. Although both urban and rural consumers predominantly access meat, fish, poultry, vegetables, and fruits from informal markets (i.e., wet market), urban consumers have closer proximity to these markets (Table 2). Fig. 3 also reveals that even if promotional elements of food are not yet pronounced in informal markets, low- and middle-income households seem to consider extrinsic quality attributes as important: the product's source or brand, that the product is sealed and features a label (in packaged format), with attractive packaging, and being prominently displayed in store. In a broader context, labeling and packaging are considered extrinsic quality attributes that reinforce a product's perceived value and quality expectations (Demont and Ndour, 2015; My et al., 2018, 2021; Custodio et al., 2019), which are mostly prominent in food items being sold in modern retails stores. Another general observation from Fig. 3 is that the majority of consumers in the urban districts consider the importance of nutrition cues on the packaging, but this still needs to be reinforced among rural households. These findings may serve as a starting point in developing nutrition interventions focusing on promoting healthier food options through labeling and packaging, even if sold in informal markets, but literacy level of target consumers and associated costs for the marketers should be carefully considered.

### 3.3 When: eating occasions

**Fig. 4:**
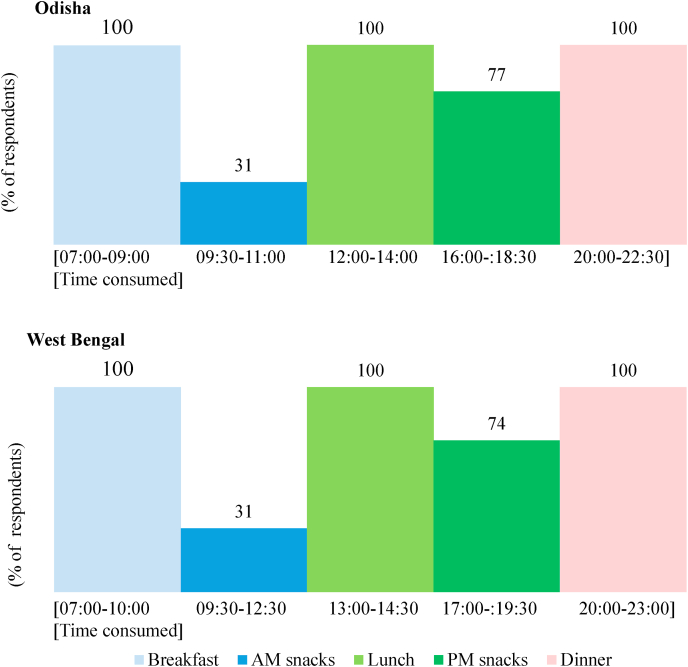
Regular eating occasions of low- and middle-income class households in the urban and rural districts of Odisha and West Bengal.

Eating occasions are another source of heterogeneity of consumers’ food choice. The regular eating occasions for households in Odisha and West Bengal are defined as breakfast, morning (AM) snacks, lunch, afternoon (PM) snacks, and dinner (Fig. 4; Samaddar et al., 2020).^1^ These are mostly consumed in-home; only about 5% of the households surveyed consumed afternoon snacks out-of-home. Since the survey focused on household consumption (versus individual consumption), household members who are left at home (e.g., housewife, elders, children) would still prepare and consume food at home, especially for lunch and afternoon snacks.

Breakfast, lunch, and dinner are considered the main eating occasions of the day. It is during dinner when most household members consume the dishes together at leisure. Eating snacks in the morning and afternoon is also found to be quite common in some households with more than 30% of households surveyed taking morning snacks in between breakfast and lunch and more than 70% of the households surveyed taking afternoon snacks in both states (Fig. 4). The latter was more prevalent among urban middle-income households. The children and office-goers, after coming back from school or office, usually eat afternoon snacks in between the longer time interval between lunch and dinner (Fig. 4). While these results suggest some heterogeneity among the frequency of eating occasions which would be relevant for nutrition interventions that target the occasion level, the largest source of heterogeneity among occasions comes from the dishes they command (Section 3.4).

### 3.4 What: dishes

**Fig. 5:**
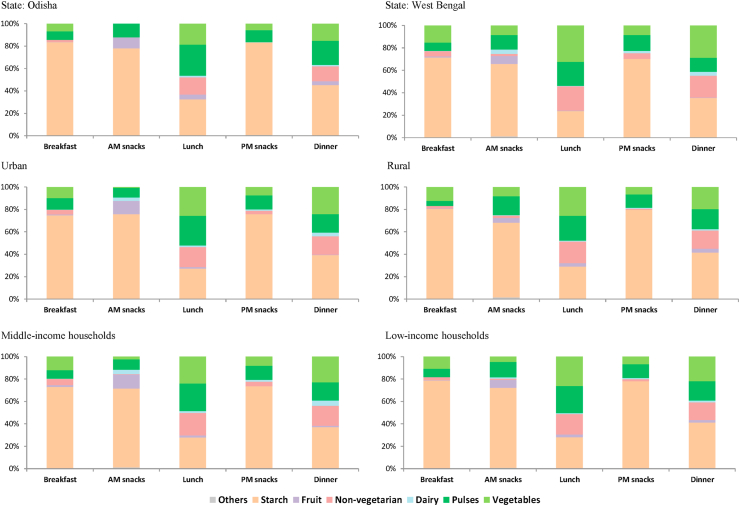
Diet diversity based on household's consumption frequency of dishes. *Notes*: For every eating occasion, respondents identified the dishes they consume in a typical month and corresponding frequency of consumption. Each dish was classified into (i) starch-based, (ii) fruits/fruit-based, (iii) non-vegetarian, (iv) dairy, (v) pulses, (vi) vegetables. The average share of frequency of consumption for each food group on a given occasion was then computed.

The dishes that consumers eat are a major source of heterogeneity of food choice. Samaddar et al. (2020) captured the diversity of Bengali and Oriya dishes through expert elicitation workshops with food experts (i.e., home scientists, nutritionists, food technologists, chefs, and restaurateurs). The resulting gastronomic system of urban and rural low- and middle-income households was validated and quantified (Fig. 5) through our survey which reveals substantial heterogeneity of food choices based on the dishes consumed as reflected in the occasion and dish levels of the gastronomic system (Fig. 1).

At the occasion level, we observed that diets are more diverse during lunch and dinner, during which non-vegetarian (i.e., meat, egg, fish, prawns), starch-based, vegetables, and pulse dishes are consumed (Fig. 5). This trend is similar between states, urbanity, and income classes. The observed diet diversity during lunch and dinner somewhat resonates the emphasis of India's food-based dietary guidelines (FBDG) on eating a variety of food (dishes) to ensure a balanced diet and access different sources of nutrients (NIN, 2011). Diet diversity during breakfast and the two snack occasions, on the other hand, is limited as households in both states mainly consume starch-based dishes (Fig. 5). This finding is consistent with previous studies by Panda et al. (2015) and Sharma et al. (2020), that indicate that Indian diets are heavy on starch and lack intake of more nutritious food.

Heterogeneity of food choices at dish level was observed with starch-based and non-vegetarian dishes (Table 3; Table 4). Differences in starch-based dishes were prominent between urban and rural households, particularly during breakfast. For breakfast, rural households in Odisha usually eat boiled corn, wheat porridge (*Dalia)* and/or *fry chura* (flattened rice) on a daily basis whereas urban households would only typically eat rice or toasted bread on a daily basis. In West Bengal, urban households consume *moori* (puffed rice), *samosa* (flour envelopes with filling), and/or fried potato on most days for breakfast. Rural households, on the other hand, consume *aloo chokha* (mashed potato with *bengal* gram)*, moori* (puffed rice), and/or boiled potato on most days for breakfast. Generally for lunch and dinner, rice (i.e., steamed, *panta bhat*) and different types of flatbread are commonly consumed in both states. Non-vegetarian dishes served during lunch and dinner vary significantly among both states with households in West Bengal preparing more non-vegetarian dishes than households in Odisha (Table 4). In Odisha, non-vegetarian dishes are mostly gravy-based such as: fish curry, egg curry, chicken curry, prawn and mutton curry whereas in West Bengal, in addition to gravy-based curries, households also eat grilled chicken (*tandoori*) or grilled fish, and beef. Pant (2010) noted that Kolkata, being the former capital of India during the British rule, has historically played a leading role in Bengali cuisine, particularly by adopting and adapting cuisine from other cultures (Table 4).

**Fig. 6:**
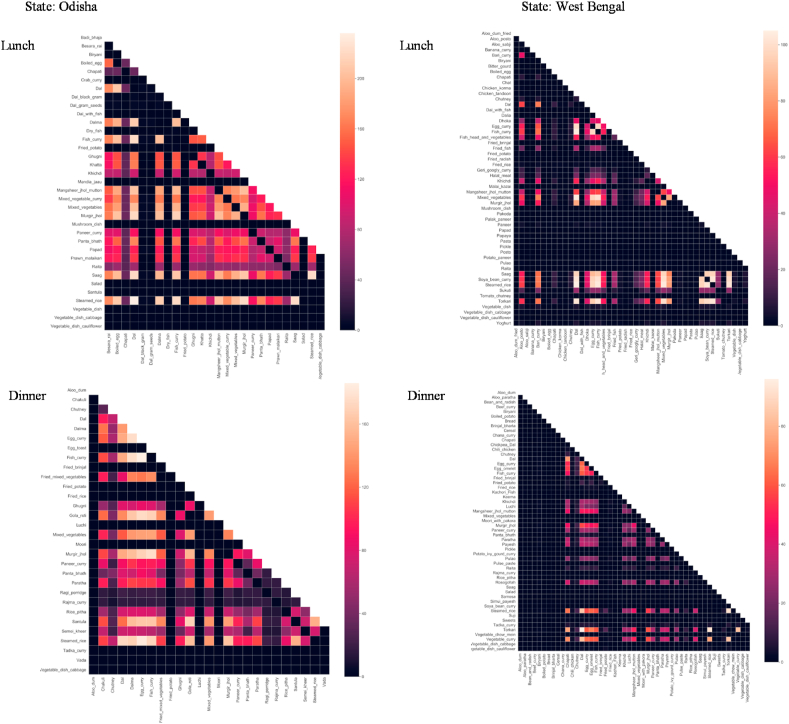
Food pairing of dishes consumed during lunch and dinner in Odisha and West Bengal. *Notes*: The heat maps visualize the co-occurrences of dishes. Co-occurrence matrices were generated by getting the dot product of two matrices such that the number of rows in Matrix 1 is equal to the number of columns in Matrix 2. The heat maps were generated based on the co-occurrence matrices of weighted frequency of consumption. Light shades represent more frequent co-occurrence of dishes. Dark shades represent less frequent co-occurrence of dishes. (For interpretation of the references to colour in this figure legend, the reader is referred to the Web version of this article.)

With a wide variety of dishes in eastern India (Fig. 5; Table 3; Table 4), we attempted to examine popular dish pairings or co-occurrences through heat maps, which revealed the critical role of rice in the diets of low- and middle income households. In Odisha, steamed rice is most frequently paired with pulse dishes during lunch (i.e., steamed rice with *dal*, steamed rice with *dalma*). Aside from the pulse dishes, pairing of steamed rice with non-vegetarian dishes (i.e., steamed rice with egg curry, steamed rice with fish curry) frequently occurs during dinner in Odisha. Interestingly, *gola roti* (a type of pancake) is also becoming a common dish during dinner in Odisha, which is consumed along with rice and paired with other vegetables and with non-vegetarian dishes (Fig. 6). Such consumption behavior is becoming prevalent as an attempt to reduce rice intake, as consumers become more health conscious while still fulfilling the satisfaction of eating rice (Panda, 2016). In West Bengal, steamed rice is frequently paired with pulses (i.e., *dal*) and with vegetable dishes (e.g., steamed rice with *torkari*, steamed rice with mixed vegetables, steamed rice with vegetable curry) during lunch and dinner.

Non-vegetarian dishes are typically consumed during lunch and dinner in both states. In West Bengal, the most frequent non-vegetarian dish pairings are steamed rice with egg curry, steamed rice with fish curry, steamed rice with *murghir jhol* (chicken curry), steamed rice with *mangsheer jhol* (mutton curry). Vegetable dishes, particularly *saag* (drumstick leaves), during lunch and *torkari* during dinner (Fig. 6) are consumed with rice prior to consumption of these non-vegetarian dishes. In Odisha, the most frequent non-vegetarian dish pairings are steamed rice with fish curry and steamed rice with *murghir jhol* (chicken curry). These non-vegetarian dishes are also paired with *saag* (drumstick leaves) during lunch and with *santula* (dried vegetable dish) during dinner. Boiled egg is also served during lunch and paired with other non-vegetarian curry dishes. Egg curry is also served during dinner and paired with *santula* and with *paratha* (a type of flatbread). Although the heat maps represent the co-occurrence of two dishes at a time, pairings of steamed rice with different types of dishes in one eating occasion indicate that a daily dining occasion usually involves combinations consisting of more than two dishes, particularly during lunch and dinner. Having these combinations of more than two dishes reflects the existence of a unique gastronomic sequence that households usually follow, which is more prominent in West Bengal (e.g., first is to consume rice with clarified butter followed by a vegetable dish with bitter taste to stimulate the digestive enzyme, then consume a slightly spiced vegetable dish which is then followed by a pulse dish, then consumption of vegetable and non-vegetarian dishes, then eating *chutney* or *papad* to cleanse the palate, and concluding the meal with a dessert) (Ray, 1987; Halder, 2016).

These food pairings further confirm that eastern Indian consumers are heavily dependent on starch, as the main source of energy derived from carbohydrates, and that rice consumption is central in their diets. Having a predominantly starch-based diet is/will be a big challenge to achieve the EAT-Lancet Commission's call for a dietary shift towards a planetary health plate, approximately half of which should consist of vegetables and fruits and the other half of whole grains (e.g., rice, wheat corn), sources of plant-based proteins (e.g., pulses), unsaturated plant oils, and modest amounts of animal-based proteins (Willett et al., 2019). The National Institute of Nutrition (2011) of the Indian Council of Medical Research reiterates the call towards a planetary health plate having considered fruits and vegetables as one of the conventional food groups in the Indian food pyramid and included in the country's Food-Based Dietary Guidelines (FBDG). This is within the premise that fruits and vegetables are rich sources of micronutrients and thus would help prevent micronutrient deficiency, which is a common nutrition problem frequently encountered among rural poor and urban slum communities. The second level of the Indian food pyramid depicts that fruits and vegetables should be consumed “liberally.”

Although it was observed in the analyses that vegetable dishes are included during lunch and dinner (Fig. 5; Fig. 6), consumption of vegetable dishes are less frequent relative to other food groups (Fig. 5). In Odisha, 19% and 15% out of all the dishes consumed during lunch and dinner, respectively, are vegetable dishes. In West Bengal, these proportions are 33% and 29%, respectively. Consumption of fruits is observed during morning snacks with 10% and 7% out of all morning snack dishes consumed in Odisha and West Bengal, respectively. Consumption of fruits/fruit-based dishes during breakfast, lunch, and dinner is minimal (Fig. 5) and is limited to fruit-based condiments (i.e., *chutney* and *khata*) (Table 3; Table 4). These findings suggest the critical need to reinforce the consumption of vegetables and fruits to respond to the call for planetary health diets.

### 3.5 Why: Consumer attitudes towards food

**Fig. 7:**
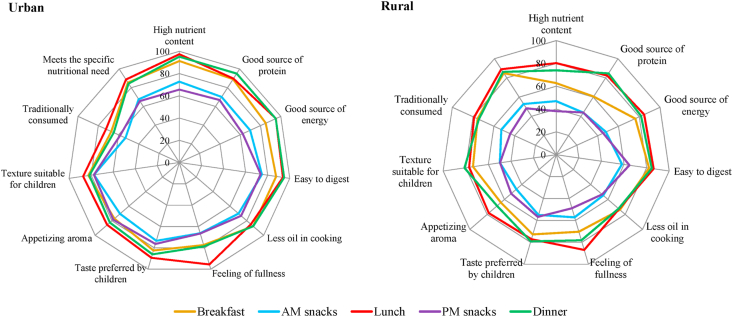
Attitude towards food of urban and rural consumers in Odisha and West Bengal based on their importance rating of 11 pre-defined attributes. *Note*: The values in the graph indicate the percent of respondents who gave a rating of 5 or 4 out of the 5-point Likert scale where 5 means extremely important and 1 means not at all important.

Heterogeneous consumer attitudes towards food are another source of heterogeneity that needs to be accounted for in designing tailored nutrition interventions, given the premise that consumers behave differently according to different beliefs, attitudes and motivations (Sheikh and Mohan 2015; Jain et al., 2014; Ajzen, 1991 Shepherd et al., 1995; Downs et al., 2020). Heterogeneity in consumer attitudes towards food was revealed in our study in three ways. Firstly, the results indicate that attitudes towards food as a source of nutrition differ between urban and rural consumers. Fig. 7 reveals that the importance of food as a source of nutrition should be examined and reinforced among rural households, who featured lower importance ratings for the nutritional attributes than urban households (i.e., high nutrient content, good source of protein, good source of energy).

**Fig. 8:**
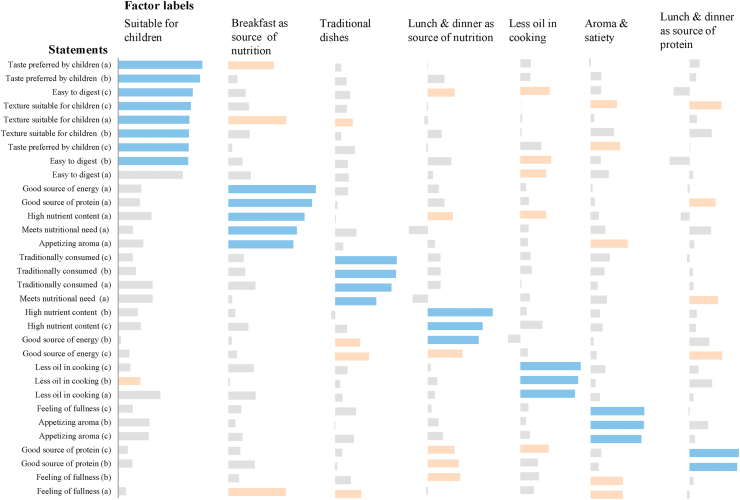
Messaging themes to increase awareness and/or promote food quality attributes based on exploratory factor analysis. *Notes*: A seven-factor solution was a result of exploratory factor analysis from 32 statements for breakfast, lunch, and dinner. The values in the graph indicate the factor loadings wherein the blue bar represents high loadings (at least 0.50), orange bar represents minimum acceptable range (0.30–0.49), and gray bars represent values < 0.30 (Hair et al., 2009). Letters in ( ) after each statement refer to the eating occasion as: (a) breakfast; (b) lunch, and (c) dinner. (For interpretation of the references to colour in this figure legend, the reader is referred to the Web version of this article.)

Secondly, consumers perceive food quality attributes differently and hence, different messages should be used to promote food based on how important these attributes are to them. Through exploratory factor analysis, our study results reveal seven messaging themes which can be exploited in designing initiatives to promote food quality attributes and in designing nutrition education campaigns (Fig. 8): (i) dishes that are suitable for children (i.e., taste, texture, digestibility), (ii) breakfast as a source of nutrition (i.e., high nutrient content, source of energy, source of protein), (iii) traditionally consumed dishes, (iv) lunch and dinner as source of nutrition, (v) less oil in cooking, (vi) aroma and satiety, (vii) lunch and dinner as source of protein.

**Fig. 9:**
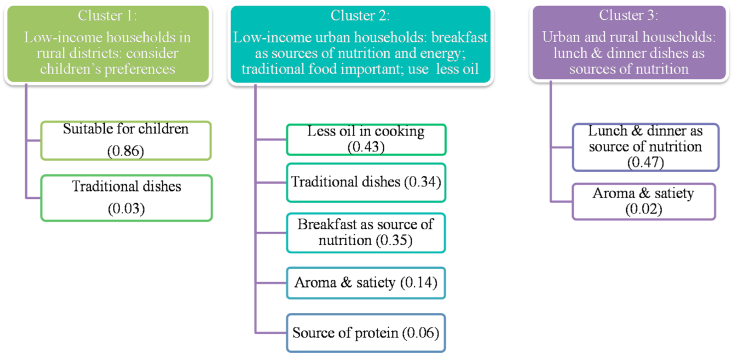
Segmentation of consumers based on their attitudes towards food quality attributes. *Notes*: The values in ( ) denote final cluster centers; computed as the mean for each variable within each final cluster and, interpreted as the characteristics of the typical case for each cluster. Non-hierarchical K-means clustering (Hair et al., 2009) was done using the factor components in the previous factor analysis as the cluster seeds. The clusters were cross-tabulated with different socio-demographic variables to provide descriptions for each cluster.

Thirdly, heterogeneity in consumer attitudes was revealed through different consumer profiles (see also Section 3.1), based on their attitudes towards food quality attributes. Fig. 9 reveals three consumer segments. We labeled these segments as (i) Cluster 1: low-income households in rural districts who consider children's preferences; (ii) Cluster 2: low-income urban households who consider breakfast dishes as sources of nutrition and energy, and who consider traditionally consumed food on specific eating occasions important, who consider reduced use of oil in cooking important; (iii) Cluster 3: urban and rural consumers who consider lunch and dinner dishes as sources of nutrition (Fig. 9). Consumers in Cluster 1 typically consider the taste preference of children, suitability of texture for children, and ease in digestion. Consumers in Cluster 2 have several considerations. Firstly, they consider food consumed during breakfast as a source of nutrition and energy. Occasion-focused messaging with emphasis on breakfast will most likely appeal to this consumer segment (e.g., *eat breakfast like a king, lunch like a prince, and dinner like a pauper*; (Kahleova et al., 2017). Secondly, Cluster 2 consumers consider reduced use of oil in cooking important. Modification of the cooking method (i.e., usage of oil) would most appeal to this consumer segment. Thirdly, it is also important for them that dishes during breakfast, lunch, and dinner feature food that is traditionally consumed during those occasions. Consumers in Cluster 3 consider food consumed during lunch and dinner as the primary source of nutrition intake.

## 4 Conclusions and implications

Unraveling the heterogeneity of food choices is critical in formulating tailored nutrition interventions to develop planetary health diets. Through a consumer survey with urban and rural low- and middle-income households in eastern India, we unraveled the heterogeneity of food choice of a target population by characterizing their gastronomic systems and examining their attitudes towards food. The three levels of the gastronomic system—occasions, dishes and ingredients—provide entry points for nutrition interventions in food environments that can nudge the target population towards healthier diets and generate nutrition and health outcomes. Our findings reveal five sources of heterogeneity (5 Ws) that affect diets—*who, where, when, what, and why*.

The first source of heterogeneity originates from the diversity of socio-economic contexts of the target population (*who*). While female household members are predominantly involved in purchase decision-making of food products for the household and in meal planning or cooking, our study results reveal that there are households where male household members are also involved in these activities and hence may also influence food choice of the households. This is important for targeting nutrition interventions.

The second source of heterogeneity of food choice is the food environment where food is accessed (*where)*. Our findings suggest that fruits and vegetables are usually purchased from weekly markets, which are usually located farther from home relative to other store types, which partially contributes to infrequent purchase. Consumers' perceptions towards the promotional element of the food environment (i.e., product's source or brand, sealed and labeled, attractive packaging, prominent store display, nutrition cues) may also be utilized as a touchpoint to promote healthier food options sold in informal markets, which may mostly appeal to urban consumers.

Eating occasions are another source of heterogeneity of consumers’ food choice (*when*) but dishes consumed during each of these occasions are the major source of heterogeneity (*what*). Our study results reveal that diets are more diverse during lunch and dinner wherein non-vegetarian (i.e., meat, egg, fish, prawns), starch-based, vegetables, and pulse dishes are consumed. Diet diversity during breakfast and the two snack occasions, on the other hand, is limited as households mainly consume starch-based dishes. Heterogeneity in dishes is prominent among starch-based and among non-vegetarian dishes. Differences in starch-based dishes were observed between urban and rural households, particularly during breakfast, while differences in non-vegetarian dishes were observed among the two states. Dish pairings or co-occurrences reveal that pairings of steamed rice with different types of dishes in one eating occasion may indicate that a daily dining occasion usually involves combinations consisting of more than two dishes. Having these combinations of more than two dishes reflects the existence of a unique gastronomic sequence that households usually follow. Heterogeneity in the consumption of dishes, the eating occasions during which they are consumed, the ingredients and ingredient pairings they are composed of, and dish preparation techniques, can provide insights into the prevalence of healthy versus unhealthy eating behavior, which can help communication programs articulate behavioral change recommendations such as dish or ingredient substitutions, modifications in preparation techniques and distribution of food intake among eating occasions. Our study findings, for example, reveal that consumption of vegetable and fruit/fruit-based dishes during breakfast, lunch, and dinner is limited. The predominantly starch-based diets featuring infrequent intake of fruits and vegetables suggest the critical need to reinforce the consumption of vegetables and fruits to respond to the call for dietary shift towards planetary health diets.

Heterogeneous attitude towards food was observed among consumers and needs to be accounted for in designing tailored nutrition interventions, given the premise that consumers act differently according to different beliefs, attitudes and motivations. Heterogeneity in consumer attitudes towards food was revealed in three ways: (i) the importance of food as a source of nutrition is more pronounced among urban consumers; (ii) seven messaging themes can be exploited in designing initiatives to promote food quality attributes and in designing nutrition education campaigns; and (iii) consumers can be segmented into three clusters which can be further used to design nutrition interventions.

Based on these findings, we recommend various nutrition interventions which generally aim at influencing consumers' personal practices and enhancing their food environment. The former can be facilitated through the three levels of consumers' gastronomic system (i.e., occasion, dish, and ingredients). The main challenge is to increase intake and/or incorporate fruits and vegetables in consumers’ starch-based diets to improve diet diversity. The general recommendation is to design nutrition promotion and education with different themes or messages specific to the profile of each segment identified in Fig. 9.

For the consumer segment that primarily considers children's preferences coming from the low-income households in the rural districts (Cluster 1), the possible message to prioritize is that fruits and vegetables are rich sources of vitamins, minerals and dietary fiber necessary for children's development, and helps prevent micronutrient deficiencies. The intervention at the occasion level may promote the afternoon snack as an occasion for children to consume fruits and provide recommendations on the quantity and types of fruits suitable for children. This may be incorporated in specific child-feeding strategies that may have already been implemented (Hodder et al., 2018). At the ingredient level, the intervention may focus on dish modification or ingredient substitution as a way to incorporate vegetables in popular children's dishes (without altering the taste).

For the consumers with a profile similar to that in Cluster 2, the possible interventions may focus on diet diversity and the role of fruits and vegetables (e.g., indigenous or traditional vegetables, locally-produced) as sources of necessary nutrients. Specifically at the occasion level, the message that may be emphasized is to consume food from different food groups (i.e., vegetables and fruits) during breakfast to boost one's energy needed for the day and to optimize nutrition intake. At the dish level, the intervention may emphasize consumption of fruits beyond *chutney* (a fruit-based condiment), increasing intake of vegetable dishes and reducing intake of starch-based dishes. At the ingredient level, the intervention may be to educate low-income household about indigenous vegetables that can be used in cooking and probably how to grow them. For the consumers with a profile similar to that in Cluster 3 who consider lunch and dinner dishes as sources of nutrition, ingredient-level intervention may be most effective, especially to reinforce consumption of fruits more than *chutney* and cucumber.

The focus of nutrition interventions to enhance the food environment may vary between urban and rural contexts. For the rural consumers, our study results suggest to prioritize (i) improvement in physical access of fruits and vegetables, which are not home-grown or those coming from different localities, by facilitating close proximity of the built environment (informal or formal markets) and investment in cold storage facilities; and (ii) scaling out initiatives (e.g., information and education campaigns) to reinforce the role of food as primary source of nutrition and focused on traditionally consumed dishes (e.g., use of indigenous ingredients, traditional dishes). For the urban consumers, our study results suggest that extrinsic quality cues are possible touchpoints to facilitate healthier food choices, particularly (i) labeling of the nutritional content of food items; (ii) strategic in-store displays (e.g., promotion of healthier food options); (iii) reliable sources or brands; and (iv) attractive packaging.

In both urban and rural settings, policy makers may consider to utilize the public distribution system (PDS) to improve access to nutritious food, as a way to enhance the food environment. Initiatives such as providing an option to reduce households’ starchy quota (i.e., rice and wheat) and opt for nutritious quota (e.g., egg, milk, fruits and vegetables provided that storage infrastructure is well-managed) may be explored. This way, consumers in the lower segment or with smaller family size will have an opportunity to access nutritious food items such as eggs and milk that could be traded off with rice volume.

Revealing the different sources of consumers’ heterogeneity of food choices and possible nutrition interventions through the gastronomic system and food environment can help policy makers and nutritionists design decentralized, segmented and targeted nutrition interventions. For example, information on socioeconomic characteristics and attitudes toward food of the target population can help fine-tune the specific messages each segment of the target population is most likely responsive to. Information on how consumers interact with their food environments can help in the design of location specific intervention strategies. Finally, detailed information on the gastronomic system of the target population can help identify entry points for behavioral change communication programs promoting dietary diversity through dish or ingredient substitution, modifications in preparation techniques and healthier planning of food intake across eating occasions. Such decentralized, targeted and segmented approach could complement centralized blanket nutrition interventions to maximize impact on nutrition and health outcomes in the population.

It is important to note that our study has several limitations and as such, results should be interpreted with caution. Firstly, our study sample is limited to urban and rural households from the states of Odisha and West Bengal, which limits generalization of the results to the overall population of eastern India. Hence, our study results represent a snapshot of food choice patterns of the target population in these two states. Secondly, similarly to the HLPE (2017) framework, availability and affordability (prices) of food items are captured in the food environment, which is framed as an exogenous factor of the gastronomic system (Fig. 1). While we captured some salient features of the food environment such as availability (e.g., physical access) and promotion, the cross-sectional nature of our survey, conducted during November–December 2017, prevented us from fully capturing seasonal variation in availability and affordability of food items in the food environment, particularly of fruits and vegetables (i.e., Table 2). Capturing the latter would ideally require running repeated surveys throughout the year with a panel of consumers. Hence, future studies can address both limitations by expanding geographical and temporal coverage of consumer surveys to capture spatial heterogeneity, seasonal fluctuations and price-driven changes in food choice. Such detailed datasets can help policy makers and nutritionists tailor nutrition interventions to specific target populations to develop planetary health diets in various contexts around the world.

## CRediT authorship contribution statement

**Marie Claire Custodio:** Methodology, Formal analysis, Investigation, Software, Writing - original draft, Writing - review & editing, Project administration. **Jhoanne Ynion:** Methodology, Formal analysis, Software, Data curation, Investigation, Writing - review & editing, Project administration. **Arindam Samaddar:** Methodology, Investigation, Resources, Writing - review & editing. **Rosa Paula Cuevas:** Conceptualization, Software, Formal analysis, Investigation, Data curation, Writing - review & editing, Visualization. **Suva Kanta Mohanty:** Methodology, Investigation, Validation, Resources, Writing - review & editing. **Anindita Ray (Chakravarti):** Validation, Resources, Writing - review & editing. **Matty Demont:** Conceptualization, Methodology, Investigation, Resources, Writing - review & editing, Supervision, Funding acquisition.

## Declaration of competing interest

The authors declare that they have no known competing financial interests or personal relationships which have, or could be perceived to have, influenced the work reported in this article.

## Footnotes

1 Eating occasions can be broadly categorized as regular and special. Regular eating occasions refer to the gatherings of household members on a daily basis wherein food is shared by the household members. It may either be consumed in-home or elsewhere. Special eating occasions are celebratory gatherings where food is prepared and shared by household members. In the context of studying heterogeneity to inform nutrition interventions, we focus on regular eating occasions.
